# Tracts in the limbic system show microstructural alterations post COVID-19 recovery

**DOI:** 10.1093/braincomms/fcae139

**Published:** 2024-05-07

**Authors:** Sapna S Mishra, Caterina A Pedersini, Rohit Misra, Tapan K Gandhi, Bas Rokers, Bharat B Biswal

**Affiliations:** Department of Electrical Engineering, Indian Institute of Technology Delhi, New Delhi 110016, India; Psychology, Division of Science, New York University Abu Dhabi, Abu Dhabi, United Arab Emirates; ASPIRE Precision Medicine Research Institute, Abu Dhabi, United Arab Emirates; Department of Electrical Engineering, Indian Institute of Technology Delhi, New Delhi 110016, India; Department of Electrical Engineering, Indian Institute of Technology Delhi, New Delhi 110016, India; Psychology, Division of Science, New York University Abu Dhabi, Abu Dhabi, United Arab Emirates; ASPIRE Precision Medicine Research Institute, Abu Dhabi, United Arab Emirates; Department of Biomedical Engineering, New Jersey Institute of Technology (NJIT), Newark, NJ 07102, USA

**Keywords:** COVID-19, post-COVID symptoms, diffusion-weighted imaging, tractography, limbic system

## Abstract

Delirium, memory loss, attention deficit and fatigue are frequently reported by COVID survivors, yet the neurological pathways underlying these symptoms are not well understood. To study the possible mechanisms for these long-term sequelae after COVID-19 recovery, we investigated the microstructural properties of white matter in Indian cohorts of COVID-recovered patients and healthy controls. For the cross-sectional study presented here, we recruited 44 COVID-recovered patients and 29 healthy controls in New Delhi, India. Using deterministic whole-brain tractography on the acquired diffusion MRI scans, we traced 20 white matter tracts and compared fractional anisotropy, axial, mean and radial diffusivity between the cohorts. Our results revealed statistically significant differences (*P*_FWE_ < 0.01) in the uncinate fasciculus, cingulum cingulate, cingulum hippocampus and arcuate fasciculus in COVID survivors, suggesting the presence of microstructural abnormalities. Additionally, in a subsequent subgroup analysis based on infection severity (healthy control, non-hospitalized patients and hospitalized patients), we observed a correlation between tract diffusion measures and COVID-19 infection severity. Although there were significant differences between healthy controls and infected groups, we found no significant differences between hospitalized and non-hospitalized COVID patients. Notably, the identified tracts are part of the limbic system and orbitofrontal cortex, indicating microstructural differences in neural circuits associated with memory and emotion. The observed white matter alterations in the limbic system resonate strongly with the functional deficits reported in Long COVID. Overall, our study provides additional evidence that damage to the limbic system could be a neuroimaging signature of Long COVID. The findings identify targets for follow-up studies investigating the long-term physiological and psychological impact of COVID-19.

## Teaser

In this cross-sectional study, authors use whole-brain tractography to investigate microstructural changes in COVID-recovered patients. Significant aberrations in four vital tracts suggest the limbic system as a potential target for SARS-CoV-2 neurotropism. This study emphasizes the need for longitudinal research on the effects of COVID on the brain.

## Graphical Abstract

**Graphical Abstract:**
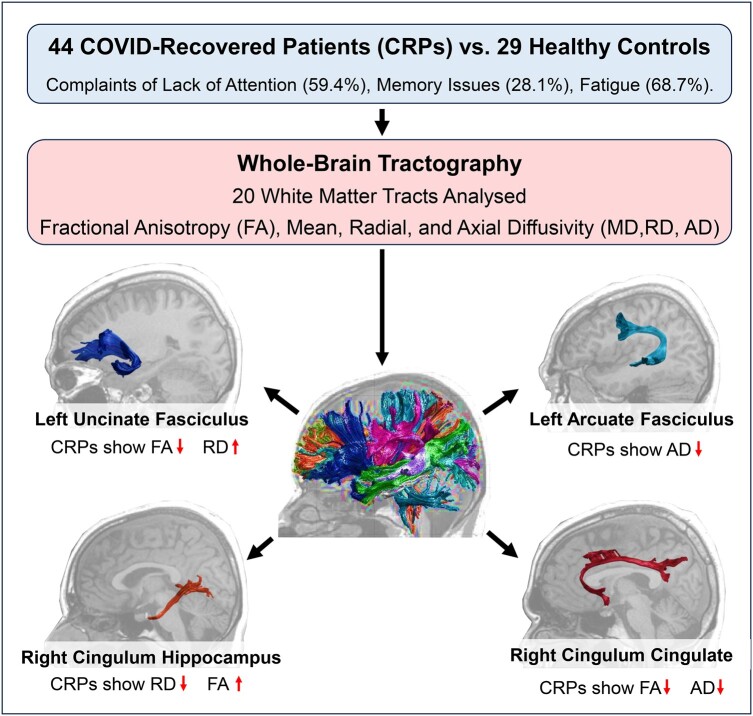

## Introduction

SARS-CoV-2 is a member of a large family of coronaviruses that causes respiratory symptoms ranging from a mild common cold to severe illnesses like severe acute respiratory syndrome and Middle East respiratory syndrome. While most casualties due to COVID-19 have been due to pulmonary complications, there are an increasing number of reports of the novel coronavirus affecting the human CNS.^1^ Recent investigations on these symptoms highlight neurological effects like memory issues, inattention, fatigue and delirium, in patients with COVID-19, even after months of recovery from the infection.^2^ The reports of long-term neurotropic effects point towards a concerning future,^3,4^ considering the large number of people that have been infected with COVID-19.^5^ This situation demands a thorough investigation into the long-term psychological, cognitive and behavioural impacts of COVID-19. Brain imaging provides one such window into the neural impact and aids neuroscientists and clinicians in the study of the neurotropic effects of the SARS-CoV-2 virus.

COVID-19 has been linked to neurological symptoms like loss of smell and taste, headaches, confusion and fatigue.^6^ Neuroimaging studies have been vital in revealing a wide array of neurological pathologies in COVID-19 patients, ranging from posterior reversible encephalopathy syndrome, leucoencephalopathy, acute infarction, cortical abnormalities and microhaemorrhages to encephalitis and ischaemic stroke.^7-11^ Even after recovery from mild infections, there have been reports of neurological issues including memory loss, extreme fatigue, attention deficit, cognitive impairment and sleep disturbance,^6,12-14^ of which the underlying mechanisms remain unclear. Upon a review of the literature, it is evident that abnormalities in the olfactory, limbic and orbitofrontal regions have been consistently reported in patients and survivors of COVID-19.^15^ A large-scale study by Douaud *et al.*^16^ investigated the morphological changes in the brain post-COVID and observed reduced thickness of grey matter in the orbitofrontal cortex and para-hippocampal areas. A multi-sequence MRI study by Yildirim *et al.*^17^ highlighted microstructural changes represented by higher anisotropy in the orbitofrontal and entorhinal cortices in COVID survivors using diffusion tensor imaging (DTI). The limbic regions and basal ganglia exhibited increased grey matter volumes and also showed significant correlations with fatigue in COVID-recovered subjects.^18^ Limbic regions including the hippocampi, Rolandic operculum, Heschl’s gyrus and the cingulate gyrus showed higher volumes in COVID survivors after a 3-month follow-up in another study.^19^ The volumetric increase in the limbic regions was also correlated with memory loss. The same study also reported a general decline in mean, axial and radial diffusivity (MD, AD and RD) in the white matter in COVID survivors along with an increase in global fractional anisotropy (FA) and significant alterations in the corona radiata, superior fronto-occipital fasciculus and external capsule.^19^ Another comprehensive multi-modal MRI study reported that cognitive dysfunction in COVID survivors showed significant association with neuroimaging biomarkers like grey matter volume and functional connectivity in limbic areas.^20^ Neuropsychiatric and cognitive tests have repeatedly reported a decline in episodic memory, attention and mild cognitive deficits in COVID survivors,^21-23^ suggesting the involvement of the limbic system in these impairments. These studies provide a refined view of the neurological impacts of COVID-19 for future investigations. The degenerative abnormalities in the limbic regions may be a signature of post-COVID syndrome. Such hypotheses are strengthened by the reports of memory and cognitive issues after recovery.

Another matter of inquiry is the possible causal mechanism for the neurological damages that persist after the infection. The symptoms may be indirect neurological manifestations of post-infections or could be a result of para-infections via pathophysiological pathways; the observations are varied.^24^ In a study,^25^ it was found that the virus had infected the CNS directly. The presence of the virus was detected in the cerebral cortex. While similar studies point to the direct neurotropic behaviour of the virus, other studies^26^ suggest otherwise. Such varied reports lead to inconclusive evidence of whether the neurological symptoms are a result of direct viral infection or are a secondary effect of physiological complications that may have occurred during the infection. In contrast to small-scale case reports, large-scale neuroimaging studies on COVID-19 patients may be able to shed light on the mechanisms behind the neurological impact of the virus in a more conclusive manner.

In this paper, we aim to use DTI to investigate the underlying changes in the nervous tissue that could have resulted in the reported neuropsychiatric effects of COVID-19 after recovery. While several studies report changes in grey matter volume,^10,18,27^ functional connectivity^28^ and magnetic susceptibility,^29^ the literature focusing on white matter tract differences in COVID patients and healthy controls (HCs) is limited. Therefore, we present a group-level cross-sectional investigation studying microstructural alterations in white matter in COVID survivors using whole-brain diffusion tensor tractography. In addition to the extensive literature reporting alterations in the orbitofrontal and limbic regions, we also observed differences in the limbic region between COVID and HCs in our prior study.^18^ Therefore, in this investigation, we hypothesized that the COVID survivors would exhibit significant alterations in the white matter tracts involved in the limbic system. Further, we also present a secondary investigation to assess the impact of the severity of COVID-19 infection on the white matter alterations, expecting to comment on whether the white matter damages are related to severity.

## Materials and methods

### Subject recruitment and demographics

For the clinical investigation and subject recruitment, a database of 2538 COVID-19 patients treated at a local hospital was employed. In this sample, 14% of patients underwent intubation (excluded from the current study), 22% required continuous positive airway pressure (CPAP), 40% received oxygen therapy and the remaining 24% did not require such assistive intervention in breathing. Two weeks after testing reverse transcriptase polymerase chain reaction (RT-PCR) negative, the patients who required CPAP (333), nasal O_2_ (333) or were admitted but did not require O_2_ (334) were investigated. We recruited subjects from this database and categorized them into the hospitalized group. Nineteen COVID-19 patients who had mild symptoms of COVID-19 were classified into the ‘non-hospitalized’ category. In addition to informed consent, the COVID-recovered cohort had to meet the following criteria to be included: (i) age > 18 years, (ii) diagnosis of COVID-19 infection based on RT-PCR test and (iii) subsequent discharge with negative RT-PCR test within 6 months prior to the scan. Eligibility requirements applied to the HC cohort were as follows: (i) subjects must be at least 18 years old and (ii) have no history of COVID-19 infection as of the scan date (if symptomatic, a negative RT-PCR test was required to confirm eligibility). Subjects were excluded from both cohorts if they had (i) history of a neurological or neuropsychiatric disorders or (ii) a history of brain surgery or trauma. Overall, in this study, we acquired diffusion-weighted images (DWIs) of 47 COVID-recovered (18 hospitalized) and 30 HC subjects. Before data analysis, three COVID subjects and one HC subject were removed during quality control assessment. Eventually, we included in the study DWIs and T_1_-weighted images from 44 COVID-recovered subjects (15 females, 35.09 ± 11.56 years) and 29 HCs (8 females, 34.45 ± 9.6 years). The details are reported in Table 1.

**Table 1:** Group-level statistics on participant demographics (whose scans were analysed) in HC and COVID-recovered groups

Measures	*stat*	*P*	Degrees of freedom	HC mean (SD)	COVID mean (SD)
Age	0.2481 (*t*)	0.8048	71	34.45 (9.60)	35.091 (11.56)
Sex	0.3427 (*χ*2)	0.5583	1	21M (8F)	29M (15F)

*P*, *P*-value; *stat*, test statistics; *t*, two-sample *t*-test statistic; *χ*^2^, chi-squared statistic; M, male; F, female; HC, healthy control.

Subjects were recruited through the Indian Institute of Technology (IIT) Delhi, and all imaging procedures were carried out there in accordance with Institutional Review Board regulations. All subjects provided informed consent before submitting any behavioural or physical information. The pilot study was authorized by the ethics committee of the Mahajan Imaging Center (internal protocol number: EC 2021/03/004), and the complete study was approved by the institutional ethics committee of IIT Delhi (project code: 2021/P019).

### MRI acquisition

T_1_-weighted images were acquired using a 3T GE scanner (Discovery MR750w) in 3D imaging mode with a fast BRAVO sequence. The scanner had a 32-channel head coil. The imaging parameters were inversion time (TI) = 450 ms; flip angle = 12°, field of view (FOV) = 256 mm × 256 mm, number of slices = 152 (sagittal), slice thickness = 1.00 mm and spatial isotropic resolution of 1 mm.

DWIs were collected using a spin-echo echo-planar imaging sequence (TR 16 000 ms, TE 76 ms, matrix size 256 × 256, FOV 262 × 263 mm, slice thickness 2 mm and voxel size 1 × 1 × 2 mm^3^) with 30 directional diffusion encoding (anterior-to-posterior phase encoding, *b* = 1000 s/mm^2^ for each direction) along with four T_2_ volumes (no diffusion encoding, i.e. *b* = 0 s/mm^2^).

### MRI pre-processing

The T_1_ volumes were thresholded to remove parts outside the brain. The cropped volumes were then segmented into different tissue types, followed by bias field correction using FSL software (https://fsl.fmrib.ox.ac.uk/fsl). FreeSurfer (http://surfer.nmr.mgh.harvard.edu/) was then used for the cortical reconstruction process of the T_1_ volumes.

DWI is an MRI technique that measures the diffusivity of water in biological tissue. It allows to derive the size, orientation, and shape of white matter fibre tracts in vivo. The diffusion MRI (dMRI) techniques exploit the diffusion of the water molecules to visualize internal physiology. In this study, the pre-processing of the DWI volumes was completed as follows. In the pre-processing of dMRI, the scans were denoised, and the warping artefacts arising due to the phase encoding direction as well the Gibbs’ ringing artefacts were removed using the MRtrix software.^30^ Further, as only one phase encoding direction of the acquisition was available to us, we utilized the Synb0-DISCO algorithm,^31^ which estimates and corrects the spatial distortions due to susceptibility-induced off-resonance fields. This step performs image synthesis to generate undistorted non-DWIs, removes non-brain tissue from the synthesized *b* = 0 data and employs the TOPUP tool of FSL. Finally, the scans were corrected for head movements and Eddy currents using the FSL toolbox.

The resulting DWI volumes were processed using the *dtiInit* function using the VistaSoft software (https://github.com/vistalab/vistasoft) in MATLAB. Here, first, the DWI scans were co-registered to the AC-PC aligned T_1_-weighted scans, and the gradient vectors were re-oriented accordingly. Further, the diffusion tensors were estimated from the re-aligned raw DWI data using a least squares fit bootstrapped 500 times. The resultant diffusion tensors were used for tractography, as described below.

### Automated Fiber Quantification

In this study, we performed tract-based analyses of the DWI data. After registration, diffusion tensors and subsequently the diffusion measures were extracted for each subject, as mentioned in the previous subsection. DWIs and structural volumes were utilized for cortical pathway tractography using the Automated Fiber Quantification (AFQ) algorithm provided in the AFQ MATLAB toolbox.^32^ The tractography algorithm identifies and extracts quantitative measures for 20 tracts in each participant’s brain. This process is completed in four steps. (i) Whole-brain streamline (STT) tractography is performed for each subject on the diffusion measure volumes while using a white matter mask to reject any stray fibres. (ii) The fibre groups are segmented into fascicles using an region of interest (ROI)-based waypoint algorithm.^30^ The identified fascicles are refined into tracts using the JHU probabilistic white matter atlas. (iii) The fibre outliers are removed from the segmented tracts to extract the core of the tract. (iv) Tract-level diffusion measures along the trajectory are calculated by employing a radial distance–weighted fibre contribution scheme. The set of diffusion measures along the tract core is defined as the tract profile. One hundred evenly spaced samples along each tract were selected to represent the tract’s profile. Finally, mean values of diffusion measures along each tract of each subject were extracted for statistical comparison. For this calculation, only the central 80 samples from the tract profile were averaged to reduce the risk of aberrant diffusion measures occurring at the interface of grey and white matter.

For each subject, trajectories and tract profiles were extracted for 20 major white matter tracts. The tracts are left and right anterior thalamic radiation, corticospinal tract, cingulum cingulate (CC), cingulum hippocampus (CH), inferior fronto-occipital fasciculus (IFOF), inferior longitudinal fasciculus, superior longitudinal fasciculus, uncinate fasciculus (UF) and arcuate fasciculus (AF), along with major and minor callosum forceps. For each tract, four diffusion metrics were determined. These include FA, MD, AD and RD.

### Statistical analysis

#### Comparison of COVID-recovered subjects and HCs

To analyse the differences between the demographics of subjects belonging to the two cohorts, we performed the unpaired two-sample *t*-test on age and the *χ*^2^ test on sex. We ran these tests on the MATLAB platform. To analyse the differences in the diffusion measures extracted from the 20 tracts previously described, we performed an ANCOVA test. In this test, the group was the variable of interest, whereas age and sex were considered covariates of no interest. To correct for multiple corrections, we performed Holm–Bonferroni correction on all four measures of the 20 tracts for a total of 80 *P*-values (20 for each diffusion measure), controlling the family-wise error (FWE) rate for *P*_FWE_ < 0.01.

#### Dependence of COVID infection on severity

Following the primary analysis, we decided to perform a secondary investigation to assess the impact of the severity of COVID-19 infection on the diffusional properties of the tracts. To analyse this effect, we categorized the 37 COVID patients based on whether their treatment required hospitalization. In this way, we formed three groups: (i) 18 hospitalized patients (HPs; 6 females, 37.89 ± 11.74 years); (ii) 19 non-hospitalized patients (NHPs; 6 females, 28.10 ± 4.79 years); and (iii) 29 HCs. As for the previous analysis, we performed an ANCOVA to compare the diffusion measures of white matter pathways between groups. Our variable of interest was group, whereas age and sex were chosen as covariates of no interest. Multiple comparison error correction was performed using the Holm–Bonferroni method over all the *P*-values (4 measures × 20 tracts = 80 comparisons) with *P*_FWE_ < 0.01. This was followed by *post hoc* tests for the tract measures that showed significant deviation in the ANCOVA test. Again, the Holm–Bonferroni method was employed to correct for multiple comparisons with *P*_FWE_ < 0.01.

## Results

In total, we recruited and scanned 44 COVID-recovered patients and 29 HCs. Thirty-seven (12 females; mean age = 33.62 ± 10.42) out of 44 COVID patients consented to disclose their COVID-19 infection-related medical information for the study. In addition, 32 (12 females; mean age = 32.62 ± 8.19 years) of the 44 patients gave permission to share their post-COVID symptoms and fatigue scores. DWI scans of all these 32 patients passed the quality check. Additionally, 19 individuals from the HC cohort (5 females; mean age = 30.63 ± 9.79 years) consented to have their fatigue scores reported. These 32 (from the COVID group) and 19 (from the HC group) volunteers were asked to rate the impact of weariness in different sectors of their daily lives (work, personal and social lives) on a scale from 0 to 5.

The severity of infection in these 37 COVID patients that consented to disclosure was categorized into two subgroups: hospitalized (18 subjects) and non-hospitalized (19 subjects). Ten of the 18 patients with severe COVID-19 infections received remdesivir, 7 required supplemental oxygen, 1 received injections of dexamethasone, ceftriaxone and clexane and 2 received bilevel positive air pressure (BiPAP). The average length of stay for the HPs was 10.78 ± 3.30 days. In these subjects, the most common symptoms during COVID-19 were fever (32/37), cough (25/37), body aches (25/37) and fatigue/weakness (9/37). Loss of sense of smell (15/37) and taste (12/37) were also frequently observed symptoms, along with difficulty in breathing (20/37).

**Figure 1:**
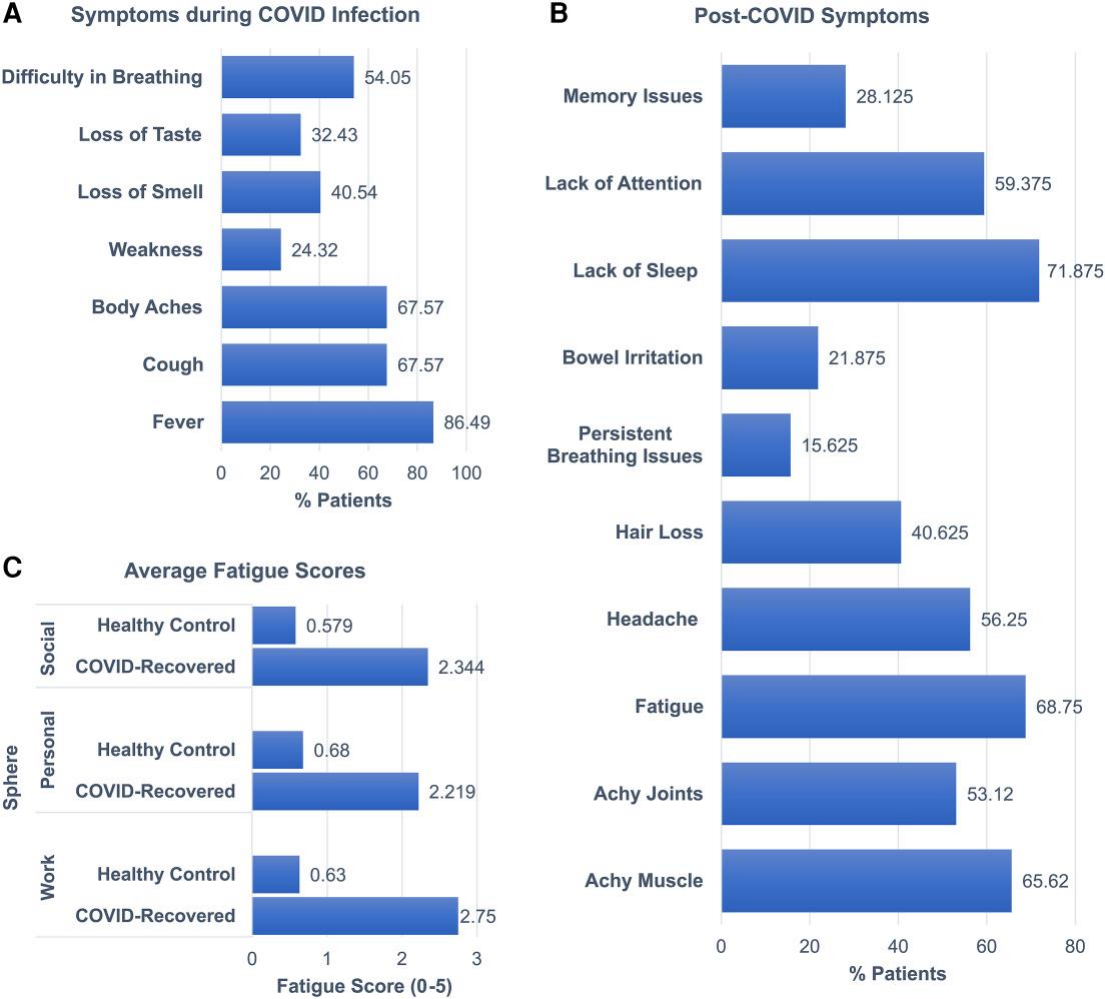
**Summary of symptoms experienced by the subjects during and after recovery from the COVID infection.** (**A**) Percentage of COVID-recovered subjects (out of 37) who reported experiencing a particular symptom during COVID-19 infection. (**B**) Percentage of COVID-recovered subjects (out of 32) who reported experiencing a particular symptom after recovery from COVID-19 infection. (**C**) Comparison of average fatigue scores between COVID-recovered cohort and HCs. The fatigue scores were reported in relation to work, personal and social spheres of life in the range of 0–5 (0 being the least and 5 being the most).

Moreover, we assessed whether these subjects had any ongoing or new symptoms from the day of discharge to the day of the scan. Thirty-two out of the 44 analysed COVID-recovered patients who agreed to share their post-COVID symptoms reported the following ailments after a negative RT-PCR test: body aches (achy muscle: 65.62% and achy joints: 53.12%), fatigue (68.75%), headache (56.25%) and hair loss (40.625%). Persistent breathing issues (15.625%) and bowel irritation (21.875%) were also specified by some of the subjects. Additionally, there were some specific complaints of lack of sleep (71.875%), lack of attention (59.375%) and issues with memory (28.125%) among these 32 patients. As mentioned earlier, these patients along with 19 HCs filled a rate on a scale of 0 to 5 on how the fatigue has affected different spheres of their respective lives: work, personal and social lives. The average scores of COVID-recovered patients on these subsets were 2.75 ± 1.244, 2.219 ± 1.361 and 2.344 ± 1.537, respectively. These scores on the HC cohort were reported as 0.63 ± 0.761, 0.68 ± 1.25 and 0.579 ± 1.26, respectively. Upon comparing the cohorts using the Wilcoxon rank-sum test, significant differences were observed in the fatigue scores for each sphere [work sphere: *P* = 9.2e^−7^; personal sphere: *P* = 1.6323e^−4^; social sphere: *P* = 1.677e^−4^]. A summary of the aforementioned self-reported symptoms and fatigue scores is provided in Fig. 1.

### Differences between patients and HCs in mean diffusion measures of tracts

**Figure 2:**
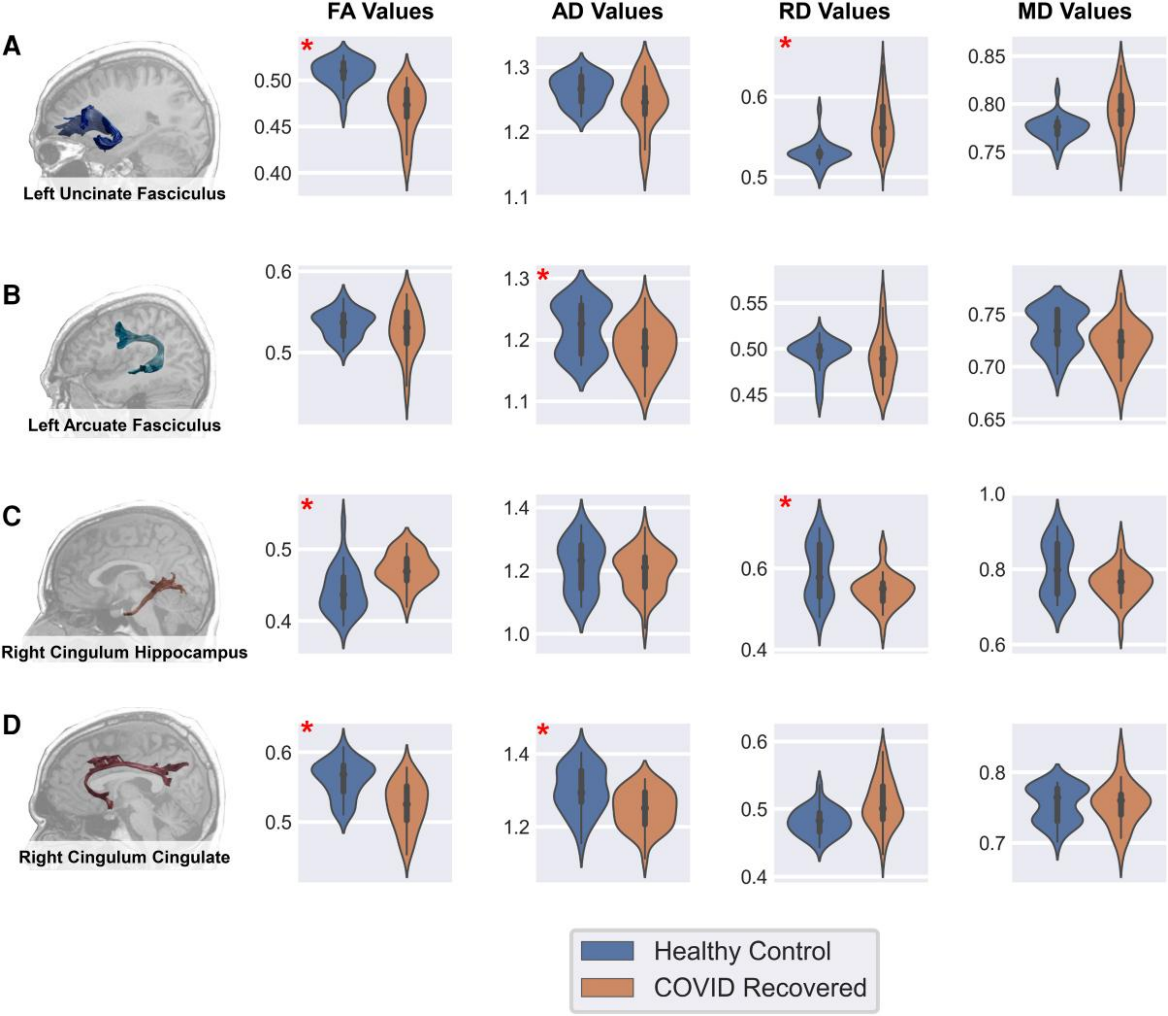
**Violin plots showing the distributions of average diffusion measures for tracts that exhibited significant differences between the two groups in the ANCOVA test.** (**A**) Left UF {FA [*F*(1,69) = 50.84, *P*_corr_ = 6.18e^−8^] and RD [*F*(1,69) = 35.14, *P*_corr_ = 8.63e-6]}. (**B**) Left AF {AD [*F*(1,69) = 17.84, *P*_corr_ = 0.005]}. (**C**) Right CH {FA [*F*(1,69) = 28.17, *P*_corr_ = 9.96e^−5^], RD [*F*(1,69) = 16.37, *P*_corr_ = 0.0099]}. (**D**) Right CC {FA [*F*(1,69) = 26.5, *P*_corr_ = 0.00018], AD [*F*(1,69) = 18.44, *P*_corr_ = 0.004]}. The tracts have been visualized in the left column overlapped to a T_1_ in the AC-PC space of one representative HC. The *x*-axis of each plot identifies the tract, grouped by cohorts HC (left violin plots in blue) and COVID recovered (right violin plots in orange). The *y*-axis represents the corresponding diffusion measures as labelled in the respective plot. The width of the plot represents the relative fraction of subjects with the corresponding value on the *y*-axis. Comparisons that showed significant differences between the groups have been marked with a red asterisk.

For each subject, we extracted 20 white matter tracts using deterministic tractography. Within each subject, we extracted mean diffusion measures (FA, MD, AD and RD) from each tract (‘Materials and methods’ section). To investigate group-level differences, we applied the ANCOVA test between the distributions of each structural measure of the COVID-19-recovered and HC cohorts for each tract, after regressing out age and gender. The results were corrected for multiple comparisons with *P*_FWE_ < 0.01. Significant changes were observed in the left UF, where the COVID cohort exhibited reduced FA [*F*(1,69) = 50.84, *P*_ucorr_ = 7.72e^−10^, *P*_corr_ = 6.18e^−8^] and increased RD [*F*(1,69) = 35.14, *P*_ucorr_ = 1.09e^−7^, *P*_corr_ = 8.63e^−6^] compared to HCs. In addition to the left UF, the COVID group showed significant changes in the right CC, where patients reported lower FA [*F*(1,69) = 26.5, *P*_ucorr_ = 2.37e^−6^, *P*_corr_ = 0.00018] and AD [*F*(1,69) = 18.44, *P*_ucorr_ = 5.61e^−5^, *P*_corr_ = 0.004] than controls. In the right CH, we observed significant changes in the FA [*F*(1,69) = 28.17, *P*_ucorr_ = 1.28e^−6^, *P*_corr_ = 9.96e^−5^] and RD values [*F*(1,69) = 16.37, *P*_ucorr_ = 0.00013, *P*_corr_ = 0.0099). Contrary to the previous results, in this tract, the COVID cohort exhibited increased FA and reduced RD. Finally, the left AF showed reduced AD in the COVID cohort as compared to HCs [*F*(1,69) = 17.84, *P*_ucorr_ = 7.21e^−5^, *P*_corr_ = 0.005]. We did not find significant differences in MD for any of the major white matter tracts. Moreover, no significant differences were observed in the other 16 white matter tracts. A summary of the statistical tests for all tract measures is provided in Supplementary Table 1. The aforementioned results for those tracts that exhibit differences between recovered patients and HCs are represented in Fig. 2.

### Relationship between diffusion measures and infection severity

The COVID cohort was categorized into HPs (18 subjects) and NHPs (19 subjects) based on whether the patients required hospitalization to treat the COVID-19 infection.

To investigate the impact of symptoms’ severity on structural measures, we performed an ANCOVA comparing the distributions of each structural measure of each tract extracted from HC, HP and NHP cohorts. In Supplementary Table 2, we report the results for all the tracts, including those that showed significant differences between groups (*P*_FWE_ < 0.01). In Table 2, we show the results of the pairwise comparisons performed on the tracts that showed significant between-group differences (*P*_FWE_ < 0.01).

**Table 2:** Pairwise *post hoc* statistical comparison of significantly different tract measures among the HCs, NHPs and HPs

Tract measures	HC versus NHP (*P*_corr_)	HC versus HP (*P*_corr_)	HP versus NHP (*P*_corr_)
FA of left UF	**0.0000**	**0.0000**	0.2152
FA of right CC	**0.0006**	**0.0005**	0.9928
FA of right CH	**0.0074**	**0.0000**	0.2412
RD of left UF	**0.0001**	**0.0000**	0.7776

Corrected *P*-values (*P*_corr_) have been presented. Tract measures with significant differences (*P*_FWE_ < 0.01) between groups are shown in bold font. 0.0000: *P*_corr_ < 0.0001.

**Figure 3:**
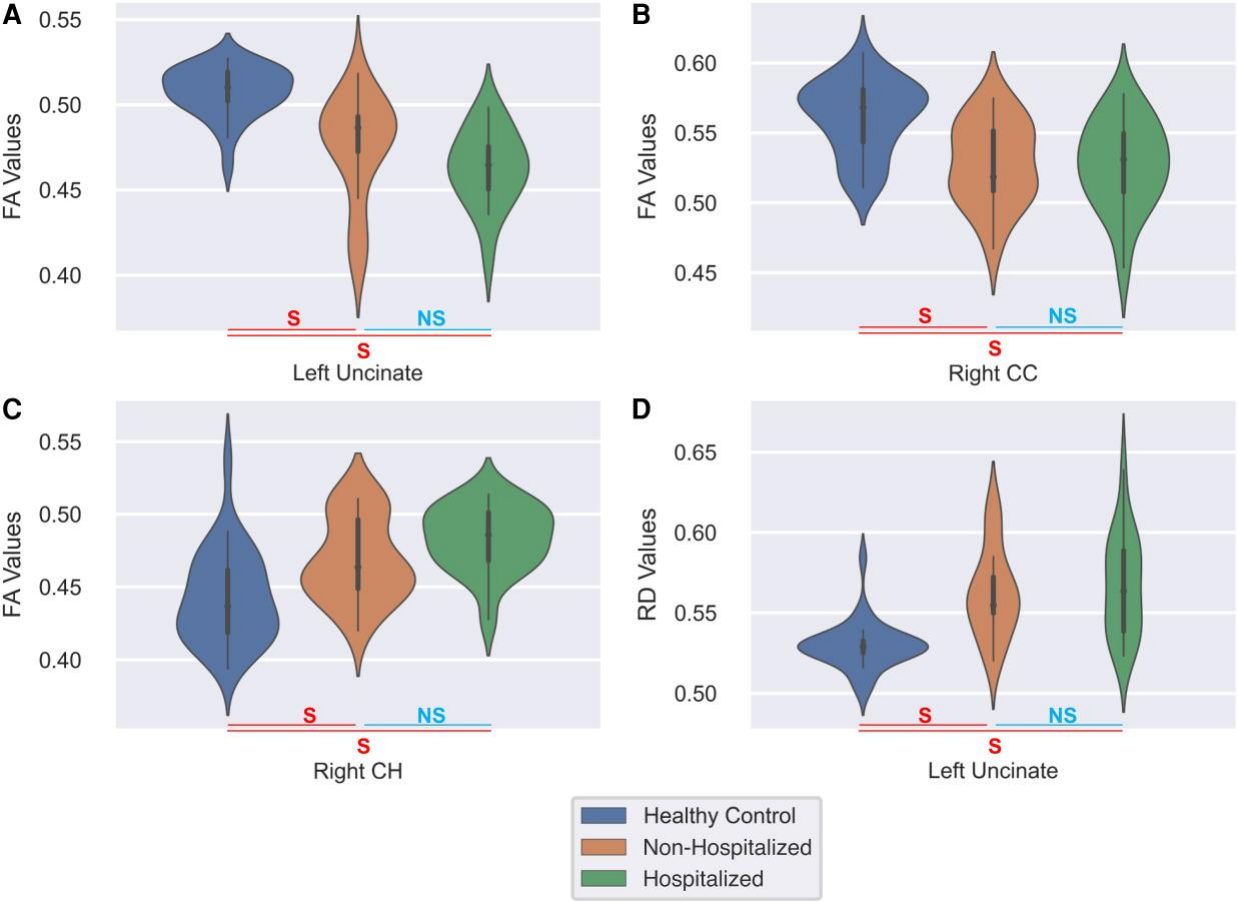
**Distributions of average diffusion measures for tracts that exhibited significant differences between the HC, NHP and HP groups are shown using violin plots.** An ANCOVA test was used to compare the diffusion measures among the three groups. FA values show a decreasing trend with severity in the (**A**) left UF [*F*(2,61) = 24.7228, *P*_corr_ < 0.0001] and the (**B**) right CC tracts [*F*(2,61) = 10.8295, *P*_corr_ = 0.0073], whereas (**C**) FA values in the right CH [*F*(2,61) = 16.0833, *P*_corr_ = 0.0002] and (**D**) RD values in the left UF [*F*(2,61) = 17.4024, *P*_corr_ = 0.0001] exhibit an increasing pattern. Further, Tukey’s honestly significant difference (HSD) test was used for *post hoc* pairwise comparison of groups. Here, the differences between the HC with NHP and HP groups were found to be significant whereas the difference between NHP and HP group was not significant. The *x*-axis of each plot identifies the tract and data grouped as HC (leftmost violin plots in blue), NHP (middle violin plots in orange) and HP (rightmost violin plots in green). The *y*-axis represents the corresponding diffusion measures where the width of the plot represents the relative fraction of subjects with the corresponding value on the *y*-axis. S, significant difference; NS, no significant difference for *P*_FWE_ < 0.01.

In this analysis, three tracts showed significant differences between the three groups, namely the left UF, the right CC and the right CH. After applying the *post hoc* tests, we found that the left UF exhibited significant alterations in FA and RD values when comparing HCs with both NHP and HP. The HP and NHP populations showed reduced FA compared to HCs (*P*_corr_ < 0.0001, *P*_corr_ < 0.0001), whereas the RD was significantly higher in both groups of patients compared to HCs (*P*_corr_ < 0.0001, *P*_corr_ = 0.0001). In the *post hoc* tests on the right CC, we observed significant alterations in FA when comparing HCs with both HP and NHP populations (*P*_corr_ = 0.0005, *P*_corr_ = 0.0006). Also, in this case, we observed a reduction of FA in both groups of patients when compared to controls. Additionally, we observed an unexpected result when considering the right CH. Indeed, in this tract, we found an increase in FA in both groups of patients when compared with controls. FA in this tract was significantly different between NHP and HCs (*P*_corr_ = 0.0074) and between HP and HCs (*P*_corr_ < 0.0001). Finally, in all the pairwise comparisons, no significant alterations were observed when comparing HP and NHP groups (*P*_corr_ > 0.01 for all pairwise comparison HP versus NHP). The aforementioned results are shown graphically in Fig. 3.

### Tract profiles of diffusion measures

**Figure 4:**
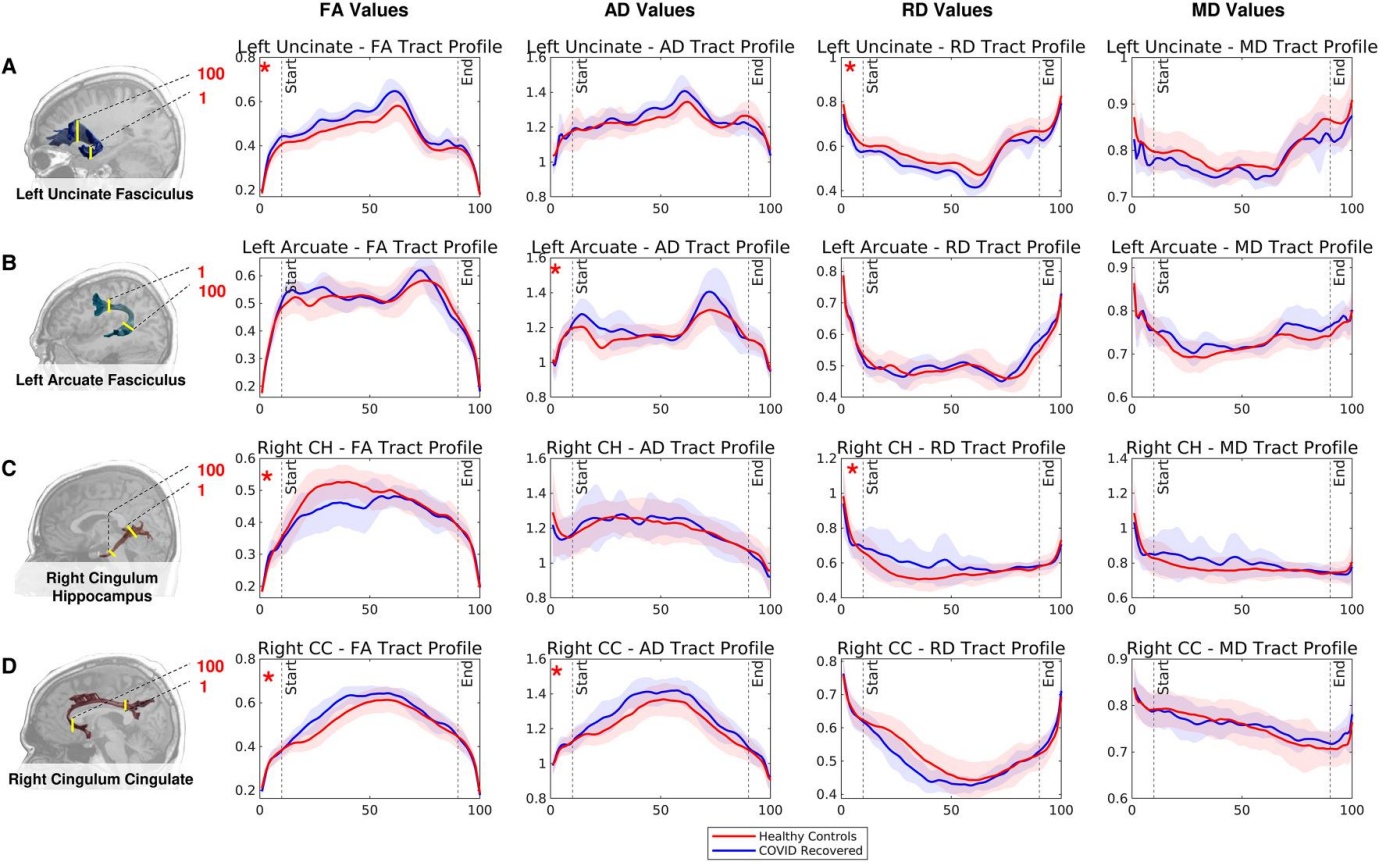
**Tract profiles of the HCs and COVID-recovered group for tracts that showed significant differences in mean diffusion measures between cohorts.** (**A**) Left UF. (**B**) Left AF. (**C**) Right CH. (**D**) Right CC. The columns represent the corresponding diffusion measure plotted, namely FA, RD, AD and MD. In the plots, the blue line represents the mean of respective measures in HCs with shaded standard deviation whereas the red line illustrates the mean of the tract profile of the COVID-recovered group along with the standard deviation represented in shaded light red regions. ‘Start’ and ‘End’ markers are shown at the 10th and 90th indexes, respectively, for each tract profile to indicate the section of the tract considered for statistical comparison. Comparisons that showed significant differences between the groups have been marked with a red asterisk.

For each structural measure and participant, we sampled the tracts at 100 equidistant points to obtain tract profiles. The diffusion measures were then averaged across participants belonging to the same cohort. For all four diffusion measures (FA, MD, AD and RD), we extracted the profiles of the tracts, which yielded statistically significant changes between the two cohorts (see Fig. 4). A consistent difference between groups in the tract profile of the left uncinate, the right CC and the left arcuate can be observed in the figure. Instead, the tract profile of the right CH shows a more pronounced between-group difference in the posterior relative to the anterior section of the pathway.

## Discussion

The cognitive difficulties experienced by COVID survivors’ post-recovery suggest long-term neurological effects of the SARS-CoV-2 virus. Multiple reports of CNS infections^2,8^ and microstructural abnormalities^33^ in COVID-19 patients warrant the need for a large-scale group-level study on the white matter tract properties of COVID-19 survivors to shed light on the neurotropic impact of the coronavirus. Hence, to perform such an analysis, we collected DWIs from 73 subjects (44 COVID-recovered subjects and 29 HCs) and extracted diffusion metrics (FA, MD, RD and AD) of 20 white matter tracts for each subject. The diffusion metrics are indicative of the microstructural properties of COVID patients’ white matter after recovery. Subsequently, we also performed a secondary analysis to investigate the effect of COVID-19 infection severity on the obtained diffusion properties of the tracts. With this analysis, we aimed to understand whether the hospitalization of patients in severe cases was a contributing factor to the white matter alterations found in the primary study.

In this investigation, we observed that certain tracts exhibited significant alterations in these properties. The left UF showed higher RD in the COVID cohort compared to HCs, accompanied by a reduction in FA. In the secondary analysis, the FA and RD values in the left UF showed a similar pattern in HC versus HP and HC versus NHP comparisons. The FA values showed a decreasing trend with infection severity, while an opposite trend was observed in RD. However, upon comparison of the NHP and HP groups, neither of these measures showed a statistically significant difference. Such alterations in diffusion properties of the tract may be attributed to demyelination or axonal degeneration in the region.^34^ Alterations in the FA of the UF have been consistently reported in COVID survivors (COVID < HC)^8,35^ and were also observed in post-COVID autoimmune encephalitis^36^ as well as other viral encephalopathies^37^ suggesting inflammatory demyelination.^37^ The left UF plays a vital role in connecting the limbic and para-limbic regions with the orbitofrontal gyrus,^38^ and disruption of the tract can be linked to problems related to emotion processing, mental health issues and the expression of memory.^39^ Our findings here directly relate to the widely reported symptoms of Long COVID like delirium, anxiety and depression.^40^

Secondly, significant differences in the FA and AD were observed in the right CC (COVID < HC). Severity analysis also highlighted significant differences for FA in the right CC in NHP versus HC and HP versus HC comparisons. Although the FA values showed a decreasing trend with infection severity, the NHP versus HP differences were not statistically significant. Such a decrease in diffusion measures may correspond to the restricted flow of ions across the axonal membranes, which could be a result of axonal injuries.^34^ Reduced FA values in association fibres,^41^ especially the cingulum, have been a common feature in post-COVID investigations,^35,41,42^ even after 3 months after discharge,^41^ highlighting the consistency of our study with independent reports. Anatomically, the CC is a prominent white matter tract that connects frontal, parietal and medial temporal sites with the subcortical nuclei.^43^ It is primarily involved in executive control functions along with emotion, pain and episodic memory.^43^ Abnormalities in the cingulum have been reported in cases of depression, mild cognitive impairment and Alzheimer’s disease.^43,44^ Similar damage to the tract may be linked to extensive reports of cognitive decline, memory issues and mental health problems in Long COVID.^40,45,46^

The right CH also exhibited significant differences between the cohorts. Surprisingly, we observed an increase in FA and a decrease in RD values in the right CH in the COVID group. The HP and NHP subgroups also exhibited significant alterations in the tract, showing an increase in FA values in their respective comparison with the HC population. While the average FA value was higher in the HPs than the NHPs, the difference was not statistically significant. Damage to the CH has been reported in independent studies on COVID-19 patients and survivors.^41^ An increase in global FA was also observed in another study on COVID survivors after a 3-month follow-up.^19^ The cingulum acts as a major pathway of the limbic system connecting the cingulate gyrus to the hippocampal formation. Damage to the tract is likely to cause impairment in hippocampal functions like learning and working memory.^44^ Contrary to some studies, our research observed increased structural integrity in patients. This conflicting data could be due to complex factors in white matter. It could reflect a reduction in extracellular volume, cell swelling, blood vessel volume changes or an increase in myelination.^47^ However, increase in FA coupled with restricted RD is typically linked with white matter maturation or increased myelination in the region.^34^ Moreover, the presence of crossing fibres, coupled with uneven decline in certain fibre bundles, along with the relative preservation of other fibre bundles apparent contradictory elevation in FA. In our case, a possible explanation for the increase in FA is that these changes are due to an increase in myelination in hippocampal and para-hippocampal areas in response to neural damage caused by the coronavirus in the other white matter tracts. Similar compensatory mechanism has been shown in the presence of Wallerian degeneration due to chronic lacunar infarcts.^48^ This axonal growth and strengthening of microstructural integrity of the tract may be a sign of the recovery process. Longitudinal follow-up with recovered patients in these and similar studies may help elucidate this question better in the future.

Finally, our study highlighted significant differences in the AD values of the left AF (COVID < HC), suggesting possible axonal injury. Aberrations in the AF have been repeatedly reported in COVID-19 patients exhibiting high RD,^42^ higher MD coupled with reduced FA,^35^ decreased FA^41^ or a general decline in AD for AF, UF, IFOF and CSP.^36^ Being a dorsal association tract connecting the frontal, parietal and temporal regions, the arcuate is generally associated with functions like working memory, social communication, language and motor coordination.^19^ Impairment of these functions can be directly linked to commonly reported symptoms of Long COVID like lack of attention, lack of sleep, persistent state of confusion and anxiety.^45,46^

It is indeed worth noting that three of the four tracts identified in this study are a part of the limbic system: namely the right CC, right CH and the UF. This system is implicated in emotion, long-term memory, behaviour and attention.^49^ Therefore, abnormalities in this system may potentially result in conditions consistent with widely reported symptoms in COVID-recovered patients, i.e. decrease in episodic memory, objective cognitive decline, weakened attention and fatigue.^21-23^ This hypothesis is strengthened by neuroimaging studies that report significant loss in limbic functional connectivity and grey matter volume.^15,18-20,28,50-53^ Microstructural alterations in the tracts involved in the limbic system, as observed in our study, could be a precursor to these functional deficits. Our study adds on to the accumulating evidence that limbic system abnormalities could be a potential biomarker in the post-COVID syndrome.^54^ This evidence can also be utilized to develop improved strategies for future studies on Long COVID.

While it is difficult to comment on the longevity of the white matter effects at this point, our secondary severity-based analysis sheds light on another important question regarding the possible mechanism of neurological effects of COVID. Varied reports on SARS-CoV-2 neurotropism have resulted in inconclusive theories of whether the neurological symptoms are due to direct viral infection in the brain or secondary manifestations of symptomatic complications.^55^ As reported in our study, the *post hoc* tests with the HP and NHP subjects showed significant alterations when comparing HCs with both groups of patients. However, no significant differences were found in the NHP versus HC or HP versus HC conditions. The diffusion measures did show a trend with severity signalling a possible link between microstructural alterations with infection severity. These findings weakly imply that the severity of the COVID infection in the subjects could be a contributing factor to the white matter aberrations observed in the differential study. On the other hand, the lack of significant differences between the HP and NHP groups disfavours the possible role of severity-linked secondary physiological complications of COVID-19 in the white matter damages observed. We believe that our findings will contribute towards the investigation of a mechanistic understanding of the neurological impact of COVID-19.

### Limitations

While this study reports important findings pertaining to white matter changes in the brain post-COVID, we identify certain factors that may be improved in future studies in the domain. The subject recruitment and MRI acquisition for the study were conducted during the early COVID-19 waves (May 2021–January 2022). Therefore, the MRI was done using standard clinical protocols prioritizing smaller scan durations for patients in critical conditions. We suggest that future studies use research protocols for acquisition with a larger number of gradient directions in DWI. Further, as the imaging for this observational study commenced after the COVID-19 outbreak, it is difficult to comment on the disease causality with certainty despite clear differences between the groups. The observed changes could be caused by the infection or, alternatively, be a causal factor predisposing certain groups to the coronavirus infection. However, a recent report of sustained subcortical myelin loss and impaired hippocampal neurogenesis in mouse models of SARS-CoV-2 infection supports the former hypothesis.^56^ Additionally, we were unable to perform any cognitive tests on the participants. Incorporating these assessments and their scores can enrich the analysis and provide better interpretations of the white matter changes in different regions of the brain. Finally, increasing the number of participants in each group along with the finer details of disease severity of the COVID-19-recovered patients can enable us to improve the generalizability of obtained results and relate the WM alterations with disease severity.

## Conclusion

The differential comparison among the HCs and the COVID-recovered cohort revealed multiple tracts in the white matter that exhibited significant changes in their diffusion properties, possibly indicating microstructural damage or injury. As hypothesized based on our prior investigations,^18,28^ three tracts of the limbic system, namely the right CC, right CH and the UF, showed significant abnormalities in COVID-19 survivors. Disruptions in the limbic system resonate clearly with the episodic memory and emotion-linked symptoms of Long COVID. Our study provides additional evidence that damage to the limbic system could be a neuroimaging signature of Long COVID.^54^ Moreover, microstructural damage in the limbic white matter could be a precursor to the widely reported functional deficits like decline in episodic memory, attention and cognitive abilities.^21-23^ In some tracts, the observed increased myelination and axonal growth may represent the result of a compensatory or recovery mechanism in response to COVID-related damage to other tracts. Moreover, the observations of this study are also consistent with neurological case studies on COVID-19 patients and group-level studies conducted using DWI and other MRI modalities. The putative functions of the tracts showing these aberrations align well with the post-COVID symptoms reported by the subjects of this study as well as in global surveys. The present investigation will aid the community in understanding and characterizing the impact of the SARS-CoV-2 virus on the brain in a better way.^57^ The study also indicates the viability of diffusion-based MRI as another tool for the analysis of post-COVID symptoms.

## Supplementary Material

fcae139_Supplementary_Data

## Data Availability

The source data are available upon reasonable request from the corresponding author. All computations were conducted with pre-existing FSL, SPM, FreeSurfer and MATLAB toolboxes as described in the ‘Materials and methods’ section. The diffusion measures extracted for each tract and subject along with the codes for analysis are available at https://osf.io/3kh59/?view_only=1796ab5dc32a4fd5be50301a52bf8a8b.
