# Supplementary material for: Tracts in the limbic system show microstructural alterations post COVID-19 recovery
Source: Brain Commun. 2024 May 7;6(3):fcae139. doi: 10.1093/braincomms/fcae139 (PMC11074789; doi:10.1093/braincomms/fcae139)
Supplement: fcae139_Supplementary_Data [file fcae139_supplementary_data.pdf]

Supplementary Material

Supplementary Table 1. Statistical comparison of Fractional Anisotropy (FA), Mean Diffusivity (MD), Axial Diffusivity (AD), and Radial Diffusivity (RD) tract measures among the Healthy Controls (HCs) and COVID-recovered cohorts. Corrected p-values (p_{corr}) and F-values (degree of freedom =1) have been presented. Tract measures with significant differences ($p_{FWE} < 0.01$) between groups are shown in bold font.

**Tract Name**	**FA**		**MD**		**AD**		**RD**	
	p_{corr}	**F-values**	p_{corr}	**F-values**	p_{corr}	**F-values**	p_{corr}	**F-values**
**Left Arcuate**	1	2.3843	0.0436	12.8263	0.0054	17.8381	1	1.0419
**Left CC**	1	1.2528	0.4025	7.5648	0.0467	12.6389	1	0.1147
**Left CH**	1	5.091	1	2.6983	1	0.3388	1	4.4067
**Left Corticospinal**	1	0.069	1	0.1108	1	0.298	1	0.5321
**Left IFOF**	1	1.3026	1	0.0291	1	0.8797	1	0.1587
**Left ILF**	0.1536	9.8673	1	2.5793	1	1.0962	0.24	8.834
**Left SLF**	1	0.045	1	0.6163	1	0.3612	1	0.4739
**Left TR**	1	4.358	1	0.0186	1	3.1446	1	1.4832
**Left Uncinate**	0.000*	50.84	0.2618	8.5846	0.24	8.803	0.000*	35.1367
**Major CF**	0.5718	6.7406	0.0138	15.526	0.0124	15.8149	0.0243	14.1989
**Minor CF**	0.1332	10.2026	0.2237	9.0142	1	0.0738	0.0401	13.0449
**Right Arcuate**	0.1811	9.4879	1	2.131	0.9936	5.6066	0.3397	7.9811
**Right CC**	0.0002	26.4972	1	0.2769	0.0043	18.4455	0.0535	12.3077
**Right CH**	0.000*	28.1696	0.3461	7.9062	1	0.4583	0.0099	16.3712
**Right Corticospinal**	1	3.6092	1	0.5198	1	1.3168	1	3.4198
**Right IFOF**	0.0535	12.2996	0.4858	7.1133	1	0.2667	0.0779	11.4192
**Right ILF**	1	3.6909	1	0.1929	1	1.2485	1	2.3743
**Right SLF**	0.4518	7.2959	1	4.0297	1	0.5418	1	5.3613
**Right TR**	0.3208	8.1334	0.5718	6.7529	1	0.6995	0.1181	10.492
**Right Uncinate**	1	3.4666	1	4.1564	1	0.1151	1	5.2138

*p_{corr}<0.0001

Supplementary Table 2. Statistical comparison of fractional anisotropy (FA), mean diffusivity (MD), axial diffusivity (AD), and radial diffusivity (RD) tract measures among the healthy controls (HCs), non-hospitalized patients (NHPs), and hospitalized patients (HPs). Corrected *p*-values ($p_{corr)}$ and F-values have been presented. Tract measures with significant differences ($p_{FWE} < 0.01$) between groups are shown in bold font.

**Tract Name**	**FA**		**MD**		**AD**		**RD**	
	**p_{corr}**	**F-values**	**p_{corr}**	**F-values**	**p_{corr}**	**F-values**	**p_{corr}**	**F-values**
**Left Arcuate**	1	0.7347	0.059	8.02	0.0435	8.4426	1	1.333
**Left CC**	1	0.2137	0.2199	6.2723	0.1204	7.0959	1	0.5296
**Left CH**	1	2.9356	1	1.5489	1	0.4744	1	2.4409
**Left Corticospinal**	1	0.7374	1	0.1392	1	0.6223	1	0.9411
**Left IFOF**	1	0.4555	1	0.5234	1	1.4173	1	0.0822
**Left ILF**	0.2707	5.986	1	3.2876	1	0.3221	0.0999	7.344
**Left SLF**	1	1.0936	1	0.7341	1	0.4329	1	0.6839
**Left TR**	1	2.219	1	2.7882	1	1.7109	1	3.0374
**Left Uncinate**	0.000*	24.7228	0.3103	5.7852	0.1399	6.8759	1.00e-04	17.4024
**Major CF**	1	2.8398	0.1765	6.5747	0.1362	6.9271	0.2717	5.9628
**Minor CF**	1	4.0091	0.2244	6.2298	1	0.4886	0.2053	6.3737
**Right Arcuate**	1	3.5277	1	0.6903	1	2.5593	1	3.1124
**Right CC**	0.0073	10.8295	1	0.7045	0.0202	9.4512	0.7451	4.7391
**Right CH**	0.0002	16.0833	1	4.1064	1	0.2745	0.0464	8.3436
**Right Corticospinal**	1	2.3565	1	0.7842	1	0.9321	1	2.7368
**Right IFOF**	1	4.3428	1	3.2183	1	0.3592	0.9461	4.4261
**Right ILF**	1	3.0029	1	0.6683	1	0.4686	1	2.6638
**Right SLF**	1	2.5038	1	1.5518	1	0.5302	1	1.7832
**Right TR**	1	3.2946	1	3.3319	1	0.6811	0.7683	4.6846
**Right Uncinate**	1	1.1715	1	2.8437	1	0.8992	1	2.3282

*p_{corr}<0.0001
